# Bridging language barriers in healthcare: a patient-centric mobile app for multilingual health record access and sharing

**DOI:** 10.3389/fdgth.2025.1542485

**Published:** 2025-02-18

**Authors:** Theodoros Solomou, Stelios Mappouras, Efthyvoulos Kyriacou, Ioannis Constantinou, Zinonas Antoniou, Ionut Cristian Canciu, Marios Neophytou, Zoltan Lantos, Christos N. Schizas, Constantinos S. Pattichis

**Affiliations:** ^1^ Department of Computer Science and Biomedical Engineering Research Centre, University of Cyprus, Nicosia, Cyprus; ^2^ HealthXR MRG Department, CYENS Centre of Excellence, Nicosia, Cyprus; ^3^ eHealthLab, Department of Electrical Engineering, Computer Engineering and Informatics, Cyprus University of Technology, Limassol, Cyprus; ^4^ National Contact Point Sector, National eHealth Authority, Nicosia, Cyprus; ^5^ Department of Virtual Health Guide Methodology, Faculty of Health Sciences, Semmelweis University, Budapest, Hungary

**Keywords:** eHealth, mobile health, patient access, medical sharing, cross-border healthcare

## Abstract

**Introduction:**

Access to health data for patients is hindered by a fragmented healthcare system and the absence of unified, patient-centric solutions. Additionally, there are no mechanics for easy sharing of medical records with healthcare providers, risking incomplete diagnoses. To further intensify the problem, when patients seek care abroad, language barriers may prevent foreign doctors from understanding their health data, further complicating treatment.

**Methods:**

Our study presents the development and evaluation of a mobile application designed to enable users to access and share their health records directly from their device, in multiple languages, ensuring ease of use and convenience. The solution utilizes OpenNCP for translating patient summaries into multiple languages and the FHIR Smart Health Links Protocol for secure sharing. We conducted a user acceptance study with 45 participants to evaluate our mobile app's interface and functionality.

**Results:**

The feedback was positive, highlighting the app's user-friendliness and usefulness. The participants felt it would enhance communication between physicians and patients and the features of sharing and translating are going to give more control of their medical data to the patients.

**Discussion:**

Based on the results and participants feedback, our mobile solution significantly enhances healthcare accessibility and efficiency by enabling easy access and sharing of health records in multiple languages, using relevant protocols and standards, reducing medical errors and ensuring personalized care.

## 1 Introduction

The European healthcare system faces significant challenges in facilitating seamless access to and sharing of patient health data, particularly across borders alongside strict regulations (1). Despite the increasing mobility of European citizens, there is a lack of unified, patient-centric solutions for sharing medical records with healthcare providers (2, 3). This fragmentation not only risks incomplete diagnoses but also potentially compromises patient safety. The problem is further exacerbated when patients seek care in foreign countries, where language barriers can prevent healthcare professionals from fully understanding patients' health data, leading to potential medical errors and suboptimal care (4–6). For instance, a tourist visiting a country where they do not speak their native language may struggle to communicate their medical history effectively, potentially resulting in misdiagnosis or inappropriate treatment. These issues highlight the urgent need for a standardized, multilingual approach to health data sharing that can overcome both technological and linguistic barriers in the European healthcare landscape (4, 7).

Our study aimed to develop and evaluate a mobile application that empowers users with access to and control over their medical data. We focused on creating a user-friendly interface with intuitive navigation and efficient functionality to ensure widespread adoption and usability. A key objective was to assess Cypriot citizens' awareness of personal health information management and to identify their specific needs in this domain. Additionally, we sought to raise awareness about the potential benefits of integrating Electronic Health Records (EHRs) into mobile applications.

In this study, we will first briefly explain the current landscape in Europe regarding mHealth, patient access, and medical sharing. Then we will examine the various components of our solution along with the system architecture. Afterwards, we will describe our pilot study design, detailing the procedures we followed and how we collected and analyzed the data. We will then present our results and insights from the pilots and finally discuss the outcomes of our study and potential future directions.

Our study aims to contribute to the ongoing development of patient-centric, secure, and efficient healthcare information systems and we are achieving that by:

1. Present and evaluate a software architecture design for implementing a national mobile health solution with cross-border sharing capabilities.
2. Demonstrate methods for ensuring accurate translation of patient summary and secure sharing of medical information
3. Highlight the needs of citizens and underscore the importance of user-centric design in healthcare
4. Provide insights into the potential impact of mobile health solutions on patient access and medical sharing.

The structure of the paper is as follows. In Section 2, the literature review is covered, focusing on mHealth applications, patient access, and medical sharing. Section 3 outlines the materials and methods, including system architecture, development tools, and pilot study design. Section 4 presents the results of the usability study and system performance metrics. In Section 5, the discussion highlights the findings, potential implications, and areas for improvement. Finally, Section 6 concludes with a summary of the study's contributions and suggestions for future work.

## 2 Literature review

### 2.1 mHealth

mHealth refers to the use of mobile devices and wireless technologies to support healthcare delivery and health management. Over the past decade, mHealth has emerged as a transformative force in healthcare, driven by the widespread adoption of smartphones, wearable devices, and mobile applications (8–10). These technologies have enabled patients to access healthcare services remotely, monitor their health in real-time, and communicate more effectively with healthcare providers (11). According to Grand View Research, the global mHealth market was valued at USD 32.42 billion in 2023 and is expected to grow significantly due to increasing smartphone penetration and rising awareness of health monitoring tools (12).

The main advantage of mHealth apps is that they significantly enhance access to healthcare, even in remote areas. This is particularly beneficial in low-resource settings, where mHealth can bridge gaps in care delivery by providing remote monitoring and telemedicine services (13, 14). For example, patients can schedule online meetings with their healthcare provider and share biometric information collected from their smart devices to help the professionals to assess the situation (15). This does not only save time for both the patient and doctor, but it also enhances their communication and offers more convenience. According to a study by Fierce Healthcare (16), the use of apps for accessing medical records increased by 50% between 2020 and 2022, highlighting the growing reliance on mobile platforms for healthcare management. Additionally, secure messaging systems within these apps enable patients to ask questions or share concerns with their doctors at any time.

Moreover, it opens up for a plethora of online interventions and features. With a smartphone you can manage chronic diseases easier since it can track symptoms, physical activity and set up reminders for the patient and studies have shown that such applications help in adherence to their therapy (17, 18). Moreover, there are mHealth solutions that offer behavioral change (19, 20) or provide mental Health support by setting up goals, gamifying interventions and offering interventions for mental health disorders, like chatbots or online forums with professionals.

However, despite its many advantages, mHealth faces several challenges that must be addressed for it to reach its full potential. One of the most significant concerns is data security and privacy (21–23). Given the sensitive nature of health information, mHealth applications must adhere to strict security protocols to protect patient data from unauthorized access or breaches. This includes implementing encryption technologies, secure data storage practices, and multi-factor authentication systems.

Finally, it is very important to mention that European governments have increasingly recognized the potential of mHealth applications to improve healthcare delivery, patient access, and cross-border medical sharing. The European Union has been at the forefront of promoting digital health solutions, with several member states adopting national mHealth strategies to enhance healthcare accessibility and efficiency. One such example is the EU4Health program, launched in response to the COVID-19 pandemic, which allocated significant funding to support digital health initiatives across Europe. With a budget of €5.3 billion for the period 2021–2027, EU4Health aims to strengthen healthcare systems by investing in digital infrastructure and promoting the development of innovative mHealth solutions (24).

### 2.2 Patient access

Patient access refers to the ability of individuals to access specific parts of their medical records, such as lab results, prescription details, and appointment scheduling, provided by healthcare organizations. A person's EHR contains comprehensive health information and should ideally be managed, shared, and controlled by the individual. However, patients currently have limited control over their EHRs, often acting more as passive observers rather than actively managing their medical data.

To address this, the European Union is enforcing stricter regulations, including the newly introduced European Health Data Space (EHDS) regulation (1), and is funding large-scale projects across Member States to build the necessary infrastructure to enhance patient access. Some of these projects include:

• **POTENTIAL** (25): Developing a national digital wallet to assist with citizen identification and storing documents such as e-prescriptions.
• **PATHeD** (26): Facilitating the translation of medical documents from one language to another.
• **xShare** (27): Creating protocol specifications and prototypes for secure and reliable exchange of medical records by patients.
• **XpanDH** (28): Defining an exchange format for healthcare resources to improve interoperability and enable cross-border sharing of medical data.
• **Xt-EHR** (29): Extending EHR capabilities to support standardized, secure, and interoperable health data exchange across member states.

As studies have shown (30, 31), patient access has numerous benefits. These include increased reassurance, reduced anxiety, positive impacts on consultations, improved doctor-patient relationships, and greater awareness and adherence to medications. Additionally, when patients have access to their data, they are better able to monitor health metrics such as blood pressure and blood sugar levels. Another advantage is that patients gain more control and flexibility in scheduling appointments with their general practitioners, which fosters stronger communication and relationships between patients and doctors.

A study aimed to compare the impact of accessing medical data through traditional methods, such as visiting healthcare providers or handling physical documents, vs. using an online portal is documented in (32). The two-year pilot involved a control group and an intervention group, with a total of around 7,000 patients. The results showed that offering the online portal led to an estimated savings of approximately $255,000, underscoring the value of patient access. Moreover, patients in the online portal group accessed their medical records significantly more frequently than those in the control group.

However, some concerns have been raised. The most significant is confidentiality, patients worry about who can access their data, as this information could affect issues regarding health disclosures, job applications, or life insurance eligibility (33). Another concern is that some results may cause anxiety if patients do not understand what they are reading due to undisclosed information, derogatory language, or inconsistencies. This is especially true when patients are diagnosed with severe illnesses like cancer.

### 2.3 Medical sharing

Medical sharing refers to the secure exchange of patient health information between individuals or healthcare providers through digital platforms like EHRs or mobile health applications. The ability to share medical records quickly and efficiently is crucial for ensuring continuity of care across different settings while improving collaboration among healthcare professionals. With advancements in digital health technologies such as Fast Healthcare Interoperability Resources (FHIR) standards (34) or Smart Health Links (SHL) (35), medical sharing has become more streamlined than ever before.

One major advantage of digital medical sharing is its potential impact on cross-border healthcare services. For instance, when a patient seeks treatment abroad or moves between countries within regions like the European Union, local doctors and healthcare organisations might not have access to the patient's national healthcare infrastructures, making them unable to access any medical information. Secure sharing mechanisms that allow the citizens to share their data with anyone they want help overcome these barriers while ensuring accurate diagnoses regardless of location.

Furthermore, especially during emergencies, quick access via shared links could prove lifesaving when time-sensitive decisions need to be taken based upon accurate knowledge about allergies/chronic conditions/medications taken regularly by unconscious/unresponsive individuals. While establishing a direct communication with emergency and prehospital healthcare support systems, such as the “AVARIS” system used by the national Ambulance Service in Cyprus (36), could enable crucial information sharing during emergency events, providing an additional tool for emergency personnel to manage incidents effectively.

## 3 Materials and methods

In this section, we will outline the technologies and components used in the development of our system and provide a detailed overview of its architecture. We will then describe the study design, including the methodologies employed and the data collection process. Each component and technology will be discussed in the context of its role within the system, highlighting how they work together to support the study's objectives. Finally, we will explain how data were gathered, ensuring a comprehensive understanding of the study's framework and its contribution to achieving reliable and meaningful results.

### 3.1 Development tool and technologies

The application was developed using React Native (37) for the frontend and C# (38) for the backend. The choice of React Native is driven by its ability to enable simultaneous development for both Android and iOS with minimal additional effort, as well as the extensive support provided by its large developer community. However, our application is communicating with multiple other components to manage the patient summary.

**Figure 1:**
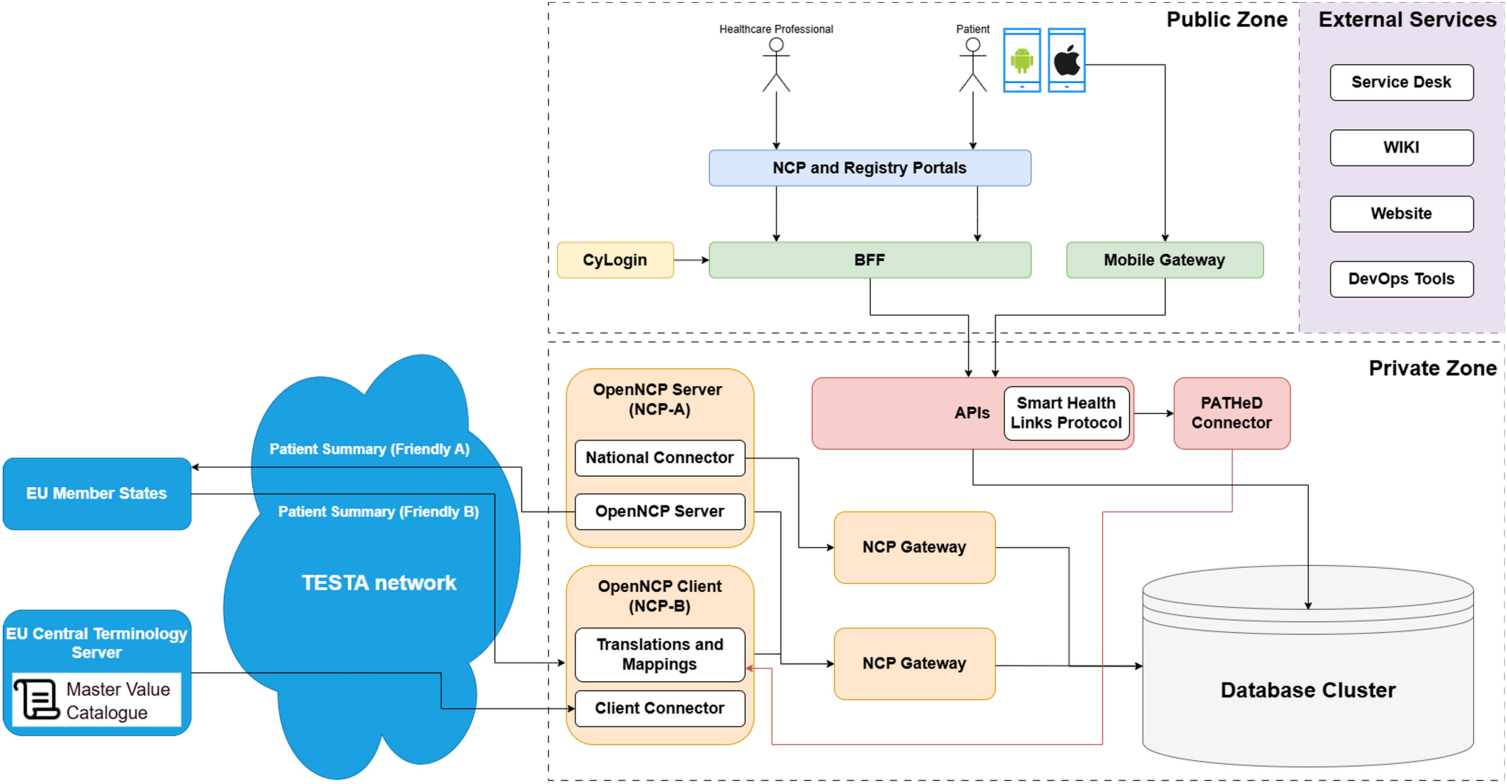
The Cyprus implementation of the CY NCPeH system infrastructure (40).

The largest and most complex system that our mobile application is communicating with is the MyHealth@EU OpenNCP system (39). In Figure 1, we can see the high-level architecture of CY NCPeH System that includes OpenNCP, which is used for cross-border health information exchange. However, due to its large complexity we will only focus on a very brief explanation of what it does and a short description of the components that we are using from this system.

NCPeH is divided into two major sections: the Public Zone and the Private Zone, which are connected through a variety of services and APIs. Its purpose is the facilitation of communication between EU member states for healthcare services, allowing interactions between patients, healthcare professionals, and external entities. In the Public Zone, the NCPeH provides healthcare professionals and patients access to services such as Patient Summary and ePrescription, which rely on the corresponding MyHealth@EU guidelines. Similarly, the Registry Portal is present, to provide information about healthcare facilities and professionals. A Backend Mobile Gateway connects mobile applications (Android/iOS), allowing healthcare professionals and patients to interact with the system. Authentication in this zone is handled by CyLogin, which serves as the login service for users. The CyLogin profile provides secure access to personal information of citizens of Cyprus, stored in government systems.

In this diagram the mobile application is making use of the Mobile Gateway, responsible for the communication between the mobile and backend services, OpenNCP-A for the patient summary retrieval, OpenNCP-B for the retrieval of the translations and mappings, the PATHeD Connector that is responsible for translating the patient summary and the Master Value Catalogue (41). The terms Friendly A and Friendly B are used in the context of the Patient Summary to indicate the language of the document. Friendly A refers to a Patient Summary that is written in the national language of the originating country, while Friendly B signifies that the Patient Summary has been translated into another language.

The Master Value Catalogue is a centralized repository that contains standardized medical terminologies and coding systems. Its primary purpose is to ensure consistent and interoperable exchange of health information across different healthcare systems and countries. The Master Value Catalogue serves as a reference for value sets, which are collections of codes and terms derived from international standards like SNOMED CT (42), ICD-10 (43), LOINC (44), and others. It enables healthcare providers in different countries to accurately understand and utilize shared health data, despite differences in language, coding practices, or local healthcare systems. By adhering to a common set of standardized codes and terminologies, the Master Value Catalogue facilitates the seamless exchange of EHRs, patient summaries, prescriptions, and other medical documents.

Additionally, we have the PATHeD Connector, an important component developed for the PATHeD project (26). Initially developed by the Hungarian team (45), this connector serves as a reference implementation, providing a blueprint for all participating pilot countries to adopt and seamlessly integrate into their national systems. Operating within backend services, the PATHeD Connector plays a pivotal role in facilitating cross-border patient data exchange. Its primary function revolves around the sophisticated handling of patient summaries, ensuring accurate and efficient translation across different languages.

Smart Health Links (SHL) (35) is a relatively new protocol that facilitates the safe and convenient exchange of healthcare data, allowing users to share their health information with anyone they choose. This protocol is designed to empower patients with greater control over their health data.

The process begins with the patient opening the application and initiating the creation of a new SHL. During this process, the user can select specific health resources they wish to share, name the link, set an expiration time, and establish an access code. Once the patient's request is submitted, our service captures a snapshot of the selected resources and saves the link configuration set by the user. The system then generates a unique, encoded link. The user can then share this link via email, open it in a browser, or present a QR code for easy access. When the recipient uses the link and enters the correct access code on our website, our service retrieves the link and displays the specified health resources.

The key distinction of SHL from traditional patient summary access is the level of specificity and control it offers. Users can choose to share precise health information with anyone, not necessarily limited to healthcare professionals who have access to patient summary systems. SHL provide versatility and can be applied in a variety of scenarios, potentially extending beyond our current anticipation. This feature enhances patient autonomy, reinforcing the idea that patients should have ultimate control over their health data. It represents a significant stride in healthcare technology, aligning with modern expectations of data accessibility and patient empowerment.

### 3.2 System architecture

**Figure 2:**
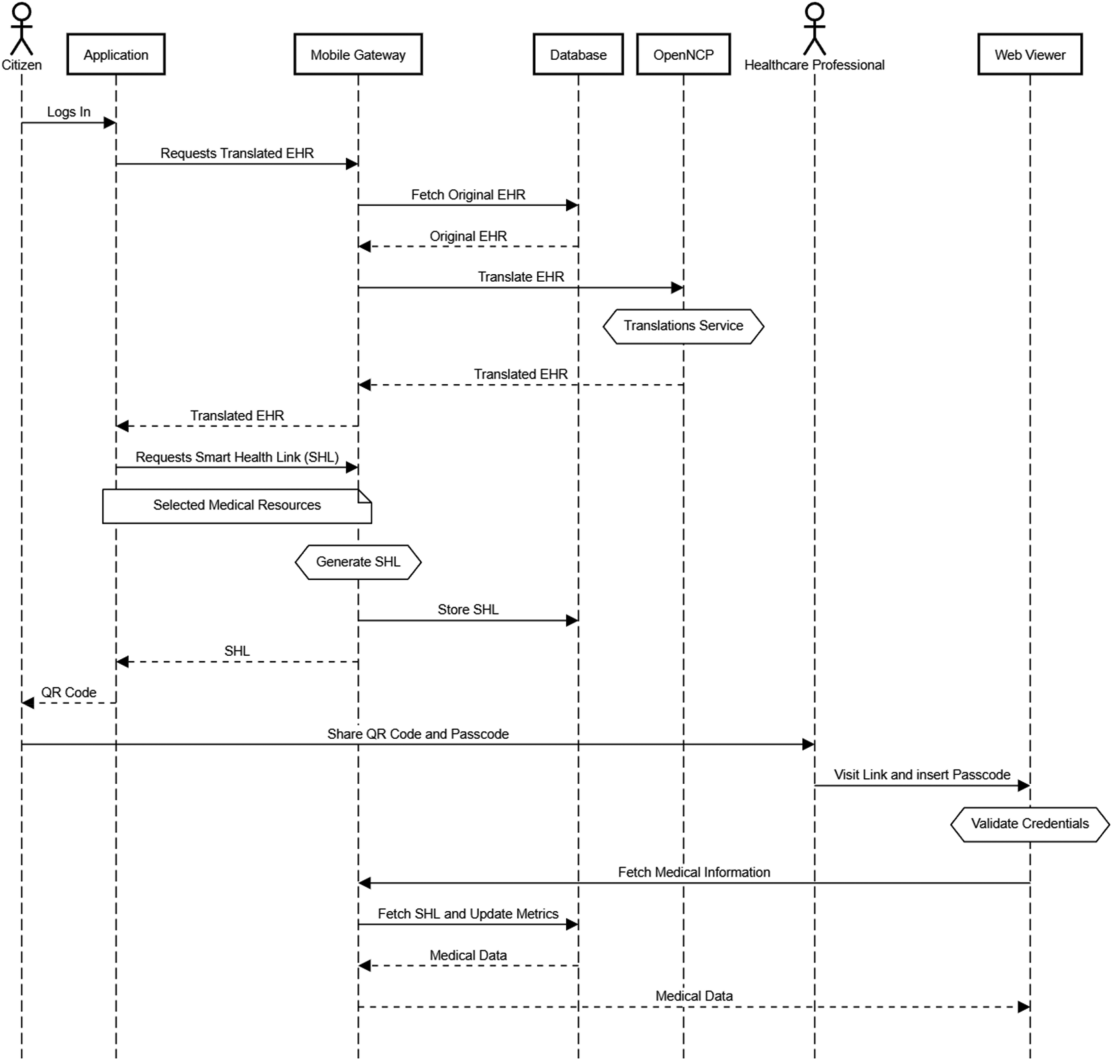
System architecture.

Our architecture consists of five main components: the Application, Mobile Gateway, Database, OpenNCP, Translation Service, and Web Viewer. Figure 2 presents a detailed diagram illustrating the interactions among these components, alongside a complete user story that demonstrates the process of translating a medical record and sharing it with a healthcare provider.

**Figure 3:**
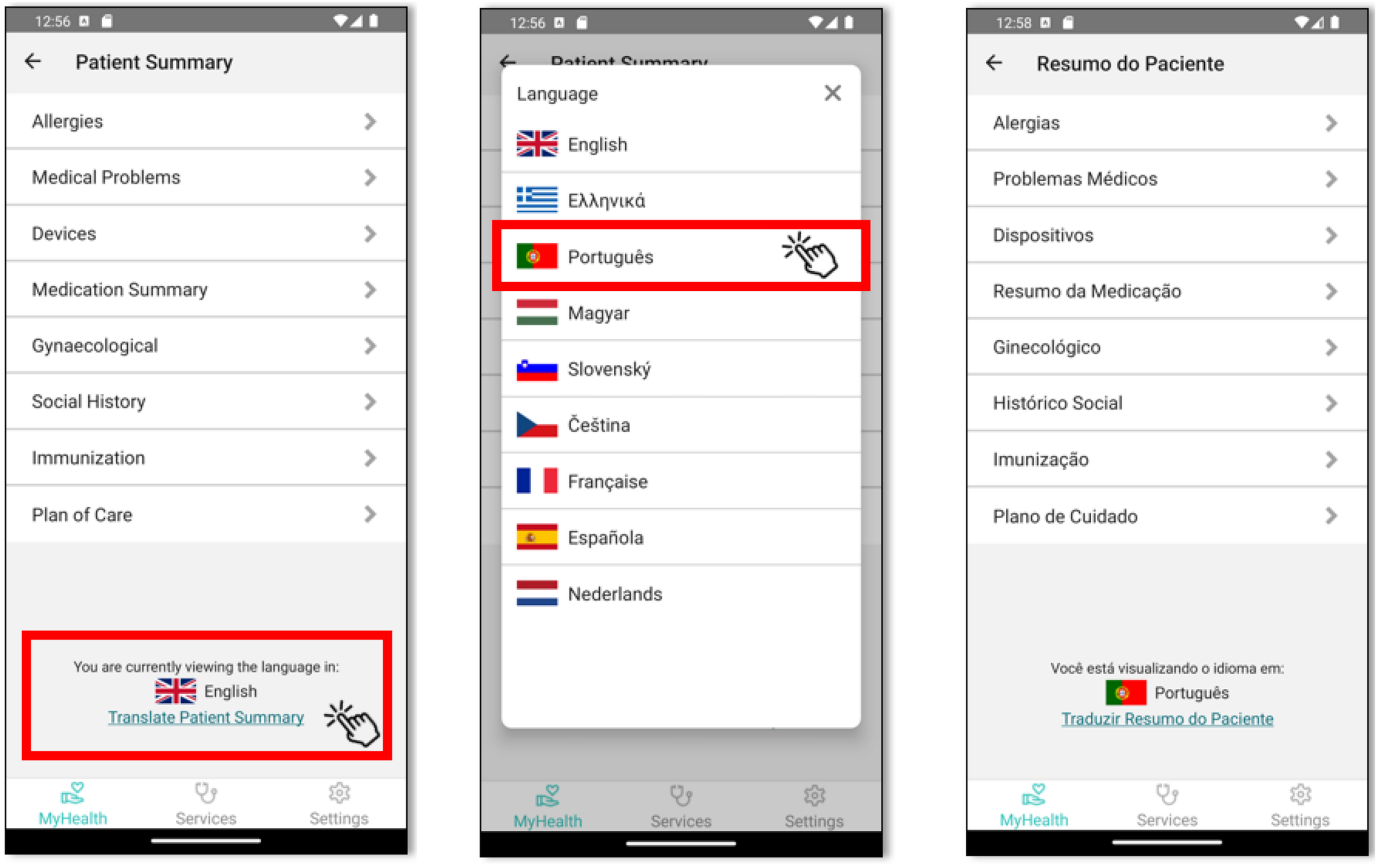
Mobile App screenshots for translating patient summary.

Our comprehensive system facilitates seamless translations and secure sharing of patient summaries across borders. The process begins when a citizen logs into our mobile application, using either a traditional username and password or biometric authentication methods such as face or fingerprint identification. Once authenticated, the user can easily navigate to their patient summary and initiate a translation request to a foreign language of their choice, as shown in Figure 3. Upon selecting the desired language, the mobile application sends a request to our mobile Gateway. This gateway then retrieves the user's original patient summary from our database and initiates an API call to OpenNCP, providing both the original document and the target language code. Within OpenNCP, the PATHeD Connector takes charge, receiving the document and expertly converting it into the specified language. Assuming a successful translation, OpenNCP returns the translated document to the mobile Gateway, which then relays it back to the mobile application for the user to view.

**Figure 4:**
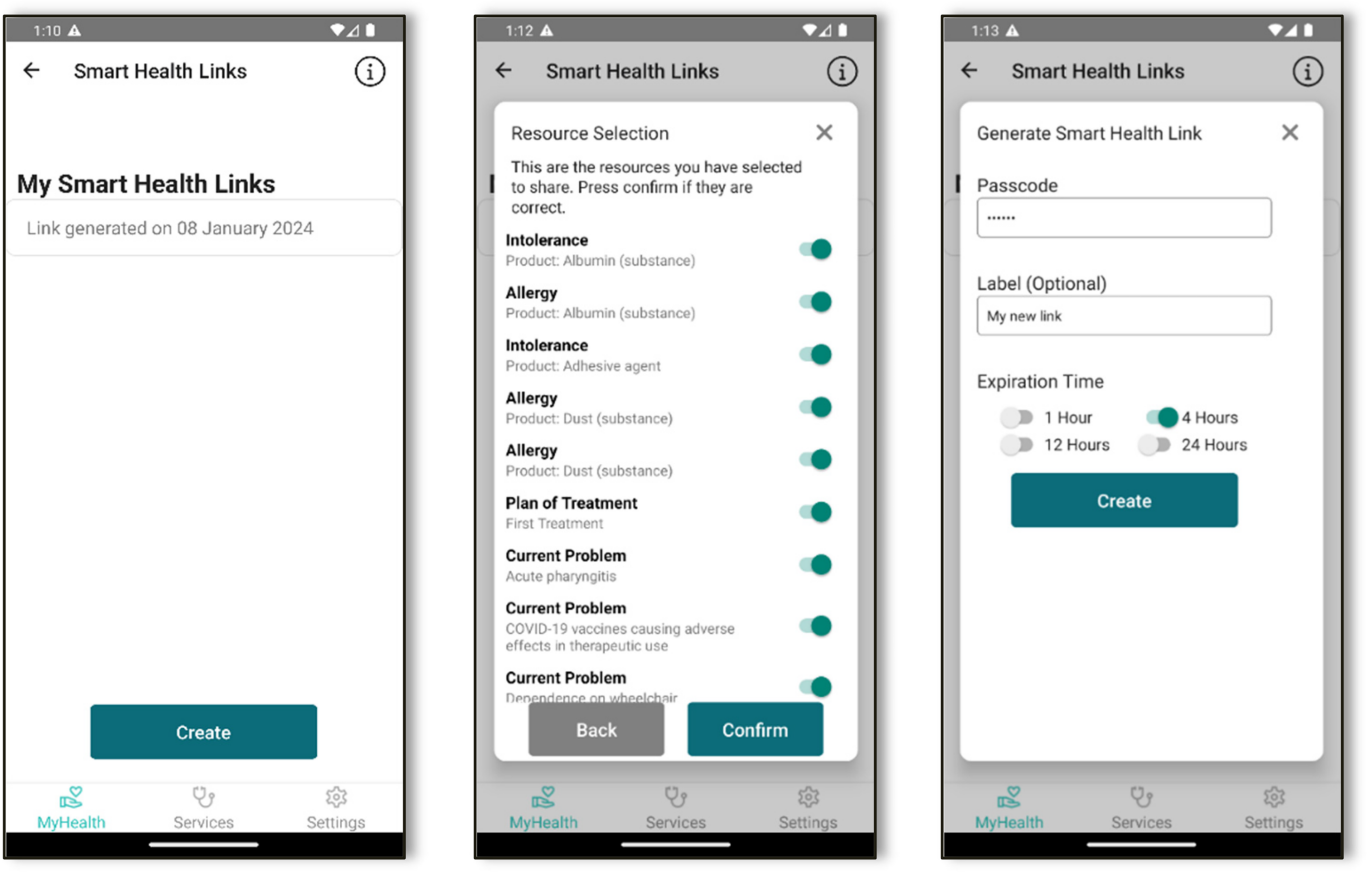
Mobile App screenshots for generating a smart health link.

With both the original and translated versions of their patient summary now, the user can proceed to share their medical information securely as illustrated in Figure 4. The system empowers users with granular control over their data sharing. They can choose to share their entire patient summary or select specific resources, depending on their preferences and the intended recipient (2nd screenshot of Figure 4). After making their selection, users are guided through a confirmation screen to verify their choices. The final step in preparing the share involves setting up a passcode, determining an expiration time, and creating a label, which are defined in the SHL protocol (3rd screenshot of Figure 4). The mobile Gateway collects this information, generates the SHL, stores the link in our database, and returns it to the user. It's important to note that an SHL doesn't contain any medical information itself; rather, it serves as a secure “guide” to accessing the data.

**Figure 5:**
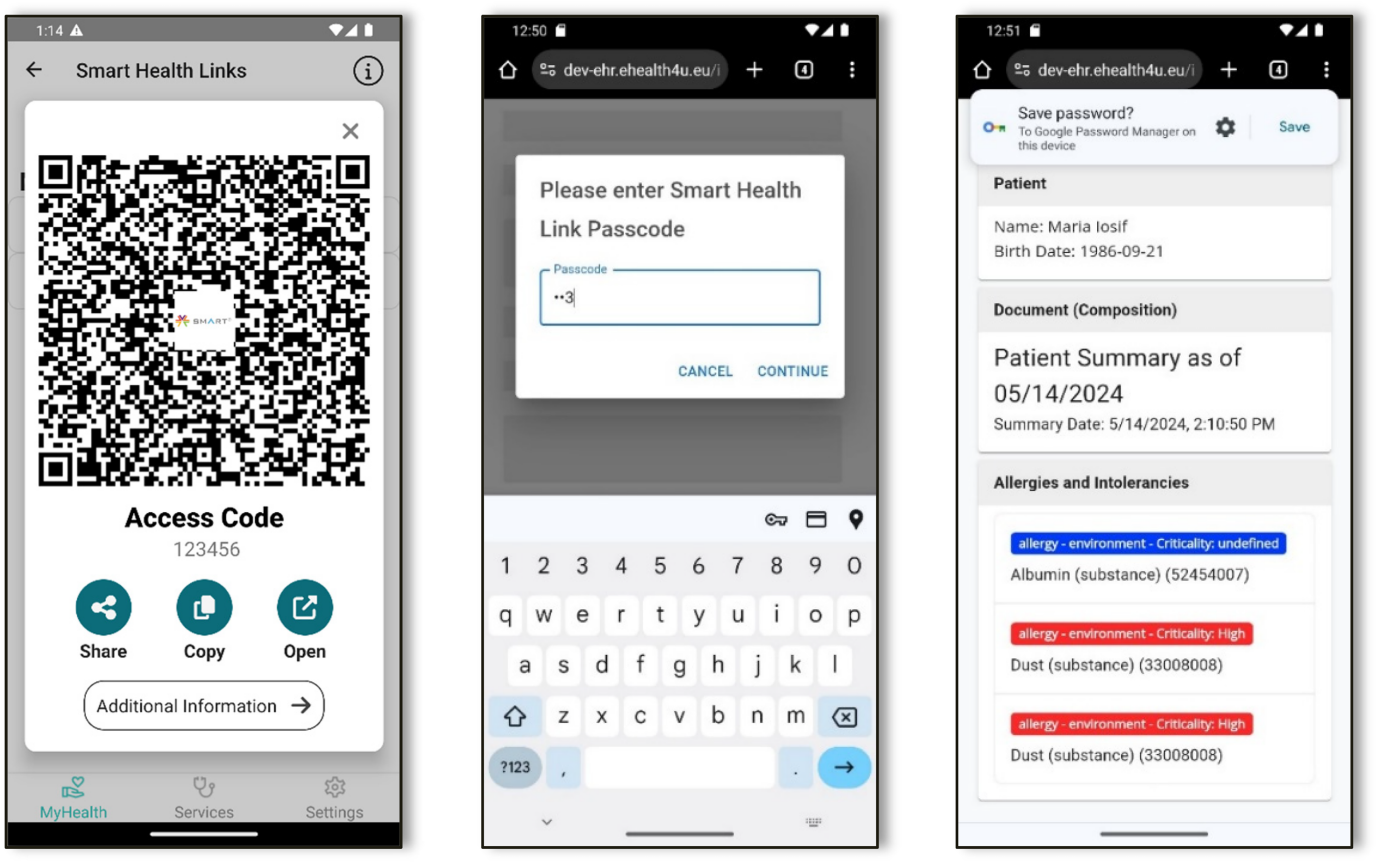
Mobile App screenshots for sharing the medical information.

As shown in Figure 5, users have multiple options for sharing the generated SHL with healthcare providers, including email, SMS, or allowing the provider to scan a QR code generated by the mobile application (1st screenshot in Figure 5). When a healthcare provider accesses the shared information through our website, our service identifies the specific information to be shared and prompts for the user-created passcode. This passcode should have been communicated separately, perhaps face-to-face or via a phone call, to ensure security.

Once the correct passcode is entered, the website communicates with the mobile Gateway to retrieve the designated medical information (2nd screenshot in Figure 5). Simultaneously, it updates the access count, logging both successful accesses and failed attempts for the specific link. If there were 5 failed attempts, the SHL is automatically blocked to ensure no brute force attacks will give access to the users' data. Finally, the retrieved data are securely displayed on the website for the healthcare professional to view (3rd screenshot in Figure 5).

### 3.3 Pilot study design

As part of the PATHeD project's requirements, six countries—including Cyprus—needed to pilot their mobile solutions using the PATHeD Connector to test its validity and integration with other national systems (26, 45). Since we were conducting our pilot to test this functionality and had already implemented the sharing feature, it seemed appropriate to extend our pilot to include these aspects and collect additional information through questionnaires. In the following subsections, we describe the design of our pilot study.

#### 3.3.1 Participants

The study included a total of 45 participants, consisting of 24 males and 21 females, with a mean age of 33.2 years (SD = 10.1 years). Marital status among participants varied, with 21 being single, 15 married, and 9 in partnership. The average number of children per participant was 0.75. Participants reported using various smartphone brands, with 11 participants using an iOS device and 34 an Android one. They were recruited by conducting workshops at local universities, the National eHealth Authority of Cyprus and the Ambulance Control Centre of the Ministry of Health. Inclusion criteria required that participants be over 17 years old.

All participants provided informed consent prior to participating in the study. No personal data were collected beyond basic demographic information such as age, gender, marital status, number of children, and smartphone brand used. Participants did not receive any monetary compensation for their involvement. Additionally, all procedures were conducted in accordance with ethical standards to ensure the confidentiality and anonymity of participants' responses.

#### 3.3.2 Procedure

In our pilot study, we explored two scenarios. In the first scenario, a tourist visiting Cyprus encountered a roadside accident. When paramedics arrived, they faced a language barrier as they and the tourist did not share a common language. For this scenario, we collaborated with the ambulance health department, where paramedics evaluated the application in practice. We conducted this scenario with eight distinct cases. The second scenario involved a Cypriot citizen traveling abroad who fell ill and required medical attention, but neither the doctor nor the patient shared a common language. This scenario included various Cypriot citizens from diverse backgrounds, representing the remainder of our cases.

Each session lasted approximately 35 min and typically included groups of four participants. During the first five minutes, the facilitator provided a brief introduction to the project, outlining its goals, explaining the problem the mobile application aims to solve, and giving instructions on how the study would be conducted. Then we had 5 min to set up the application, 10 min for navigating the app and 10 min in total to answer a pre- and post-questionnaire. Participants were then asked to sign the consent document if they agreed to proceed with the study. Due to time and hardware limitations, we were able to provide only the Android version at that time. For participants who had an iOS device, we ensured they could still participate by providing them with an Android device.

After the brief explanation and study setup, we provided the participants with a pre-questionnaire that included demographic information, their knowledge about the patient summary, what they value most in a healthcare mobile application, and other relevant questions. Upon completion, we prompted the users to scan a QR code to download the APK file of the application. We assisted them in installing the mobile application, and once all participants were ready, we supplied them with a specific account already populated with simulated data.

To allow participants to explore all the functionalities of the application without directing them explicitly on what to press or where to navigate, we designed specific tasks to guide them through the entire application with minimal interference from the researchers. The five tasks were:

- Task 1: Find the Patient Summary Screen
- Task 2: Navigate through all Screens containing the Medical Data of the user
- Task 3: Translate the Patient Summary to another language
- Task 4: Create a Smart Health Link with some constraints. E.g., only allergies or everything except allergies
- Task 5: Access the Smart Health Link resources in the browser of the smartphone.

The average time to complete all tasks was approximately 10 min, and all participants were able to complete them successfully. After finishing the pilot, the participants answered a post-questionnaire. If they had additional questions or feedback, they could discuss them with the researchers during this time. Finally, the researchers thanked the participants for their time and concluded the session.

#### 3.3.3 Data collection

In our study, we collected both qualitative and quantitative data to comprehensively evaluate the mobile application. The qualitative data were primarily obtained from the pre- and post-questionnaires that assessed users' interactions with the application. Quantitative data, mainly consisting of performance and validation metrics, were collected from our servers when API calls were triggered by the mobile application. We employed four systematic questionnaires, each designed to measure a different aspect of our application.

• System Usability Scale (SUS) (46)
  The System Usability Scale (SUS) is a reliable, ten-item questionnaire developed by John Brooke in 1986 to assess the usability of a system or product. It provides a quick and easy method for evaluating a wide range of interfaces, including websites, software, mobile applications, and hardware devices. Participants rate their agreement with each statement on a five-point Likert scale ranging from “Strongly Disagree” to “Strongly Agree”. The SUS covers aspects such as ease of use, complexity, and confidence in using the system. Scores are calculated to yield an overall usability score ranging from 0 to 100, where higher scores indicate better usability. The SUS is valuable for its simplicity, reliability, and ability to benchmark usability across different systems.
• The Single Ease Question (SEQ) (47)
  The Single Ease Question (SEQ) is a one-item metric designed to assess the perceived difficulty of a task immediately after completion. Participants are asked: “Overall, how difficult or easy was the task to complete?” They respond using a seven-point Likert scale ranging from “Very Difficult” to “Very Easy”. The SEQ provides a quick snapshot of task-specific usability without burdening participants with lengthy questionnaires. The simplicity of the SEQ makes it an efficient tool for gathering immediate, task-level feedback.
• User Experience Questionnaire – Short Version (UEQ - S) (48)
  The User Experience Questionnaire - Short Version (UEQ-S) is a concise tool designed to efficiently evaluate the user experience of interactive products. It condenses the original 26-item UEQ into an 8-item questionnaire while maintaining reliable measurement properties. Each item consists of a pair of contrasting adjectives—such as *complicated/easy* or *dull/exciting*—rated on a seven-point scale.
  The UEQ-S measures two primary dimensions: Pragmatic Quality and Hedonic Quality. Pragmatic Quality reflects the usability aspects of the product, assessing how effectively users can achieve their goals using the product. Hedonic Quality captures the emotional and experiential aspects, evaluating how enjoyable and stimulating the product is to use. By focusing on these core dimensions, the UEQ-S provides a quick yet comprehensive overview of the user's experience. Its brevity reduces participant fatigue and is particularly suitable for studies where time is limited or when integrating multiple questionnaires.
• Net Promoter Score (49)
  The Net Promoter Score (NPS) is a metric used to gauge customer loyalty and satisfaction based on a single, straightforward question: “How likely are you to recommend this product or service to a friend or colleague?” Participants respond on an 11-point scale ranging from 0 (“Not at all likely”) to 10 (“Extremely likely”). Based on their responses, customers are categorized into three groups: promoters (scores of 9–10), passives (scores of 7–8), and detractors (scores of 0–6). Promoters are loyal enthusiasts likely to repurchase and refer to others. Passives are satisfied but unenthusiastic customers who are susceptible to competitive offerings. Detractors are unhappy customers who may impede growth through negative word-of-mouth.
  The NPS is calculated by subtracting the percentage of detractors from the percentage of promoters. The score ranges from −100 to +100, with higher scores indicating greater customer loyalty and satisfaction. A positive NPS (above 0) is generally considered good, while a score of 50 or above is excellent. The simplicity and predictive power of the NPS make it a popular tool for organizations to measure customer experience, assess the likelihood of customer retention, and predict business growth.

Regarding quantitative data we collected:

• Mean response time of translating the patient summary
• Mean response time of generating a SHL
• Number of successful translations
• Number of failed translations
• Number of generated SHL
• Number of translated patient summaries that passed/failed the validation of the CDA format.

### 3.4 Data analysis

The questionnaire data analysis and results visualization were carried out using Python (50) with the Pandas framework (51) in the Google Collab environment (52). To interpret responses from the systematic questionnaires, we followed the specific scoring formulas provided within each questionnaire, ensuring an accurate interpretation aligned with its guidelines. For additional data, we employed statistical analysis techniques and clear visualizations to effectively communicate key findings.

In our data analysis process, we encountered no missing data in our questionnaires or performance metrics, eliminating the need for data imputation or other corrective measures. The bulk of our data originated from structured questionnaires, which we processed according to their respective formulas. For the open-ended questions requesting additional features and comments, we employed a qualitative approach. Each response was individually examined and categorized into specific feature requests, allowing us to identify trends and priorities among user suggestions. To visualize our findings effectively, we leveraged the capabilities of Seaborn (53) and Matplotlib libraries (54). These powerful Python tools enabled us to create clear, informative graphical representations of our data, facilitating deeper insights and more accessible presentation of results.

## 4 Results

In this section we document the results of the evaluation of the four major areas of our mobile application. First, we assess the knowledge and expectations of Cypriot citizens concerning the management of their health. Subsequently, the remaining focus areas center on the mobile application, evaluating its usability, performance, and overall participant feedback.

### 4.1 User awareness and expectations

**Figure 6:**
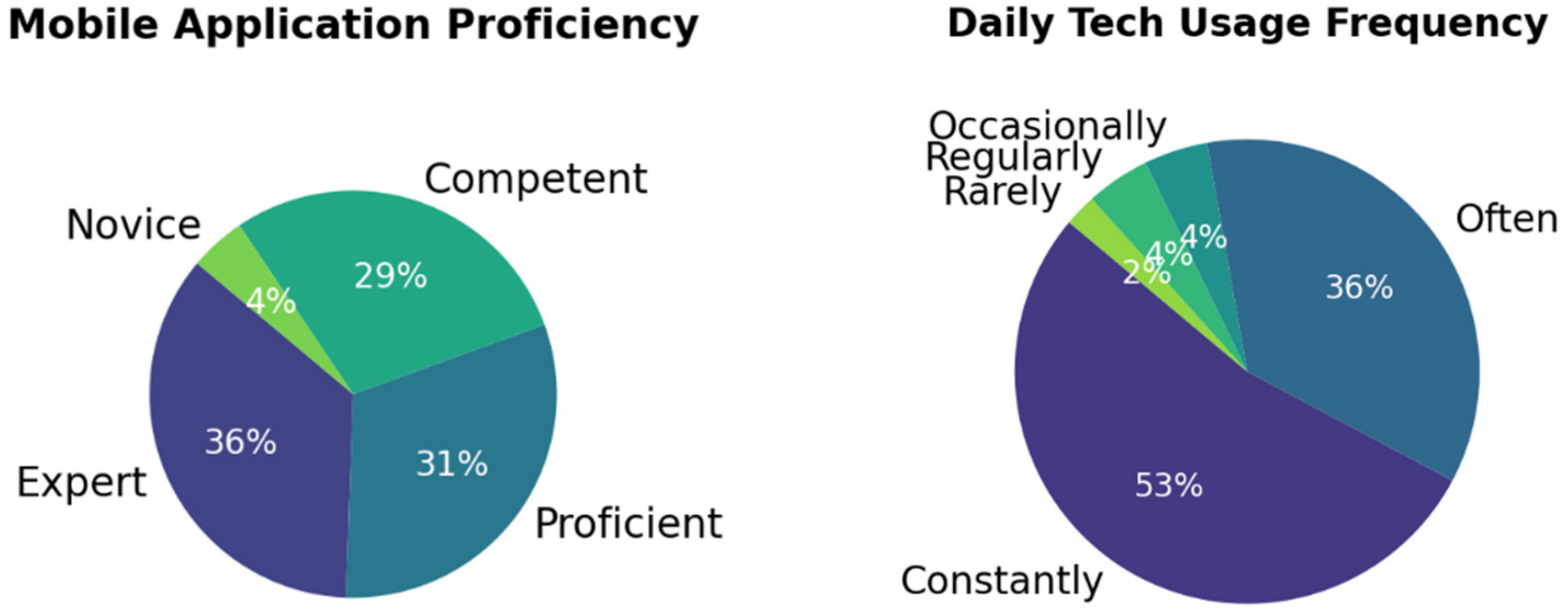
Familiarity of participants with mobile applications (left) and usage of technology in their daily life (right).

Our study began by examining participants’ familiarity with mobile applications and their use of technology in daily life. As shown in Figure 6, the first pie chart reveals that most participants have a very strong knowledge of how to use mobile applications. Additionally, most participants reported that they either explore applications independently to learn their features or seek guidance through online tutorials. The second pie chart in Figure 6 demonstrates that most participants frequently use technology to complete everyday tasks, indicating that our participant pool is familiar with mobile applications and adept at handling technology.

**Figure 7:**
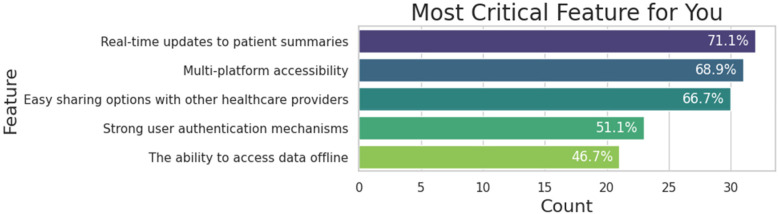
Preferred features of participants to manage their health.

Moving on, Figure 7 illustrates participants’ preferences for features in an EHR mobile application. Before interacting with the application, participants were asked to select which features they would like to have. The results show that the most popular feature is real-time updates to patient summary, which is crucial for ensuring that both patients and doctors can view new entries in the patient summary as soon as they are added. Additionally, participants expressed a strong desire for multi-platform accessibility, including websites and tablets, which would enhance the accessibility of healthcare management and not limit users to the smaller screens of smartphones.

**Figure 8:**
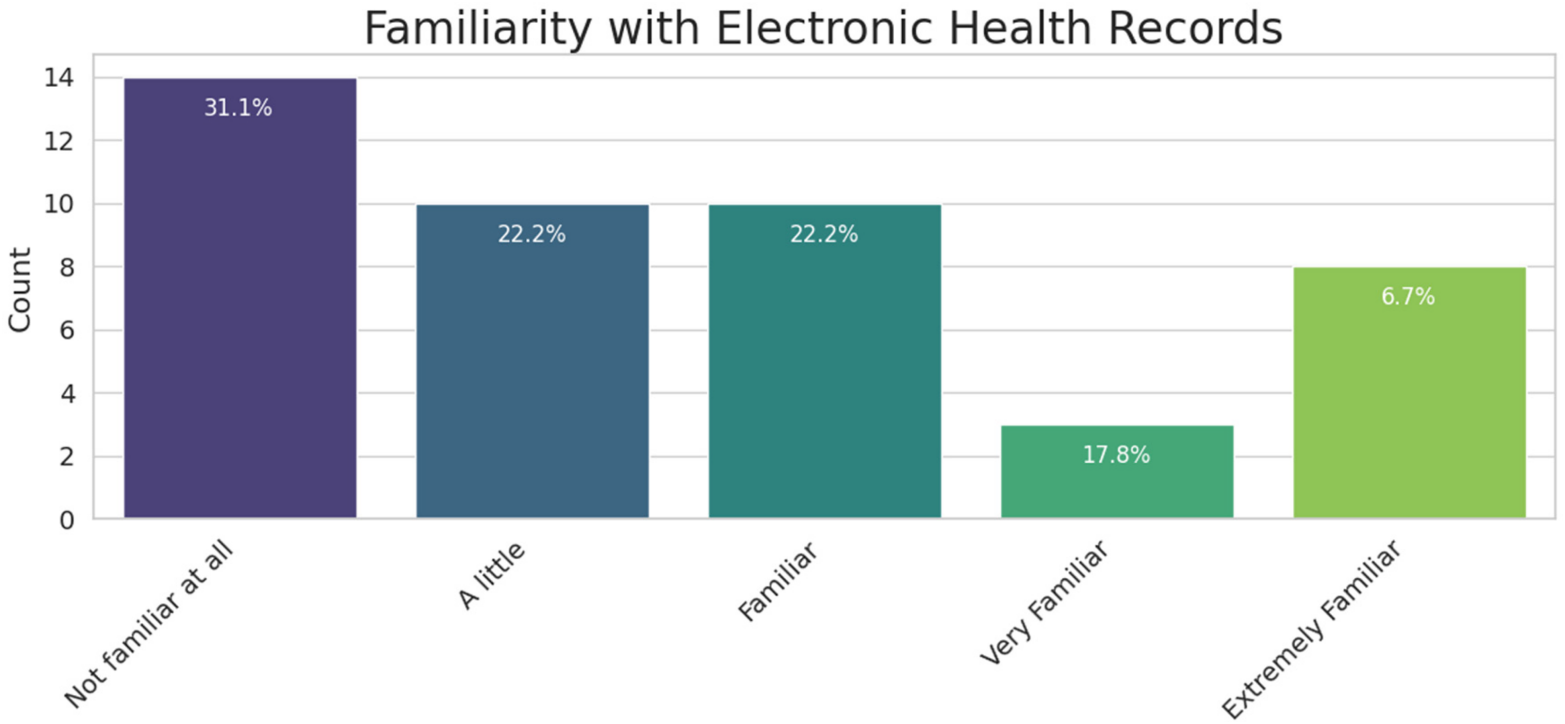
Participants’ familiarity with the EHR.

The next crucial aspect we investigated was participants' familiarity with EHR. If users are unaware that their patient summary can be accessed electronically and are unfamiliar with the collective efforts of European countries to provide this service, they might not recognize the need for these features until they consider them. Based on Figure 8, we can clearly see that participants were largely uninformed about EHRs until they participated in our pilot study. This finding highlights the need for campaigns to inform citizens about these new initiatives, enabling them to make informed decisions about whether to use such solutions. A related question asked participants how frequently they would use the mobile application, with 80 percent responding that they would expect to use it once or a few times a month. This usage pattern aligns with typical scenarios such as doctor visits, travel preparations, or occasional health status checks.

**Figure 9:**
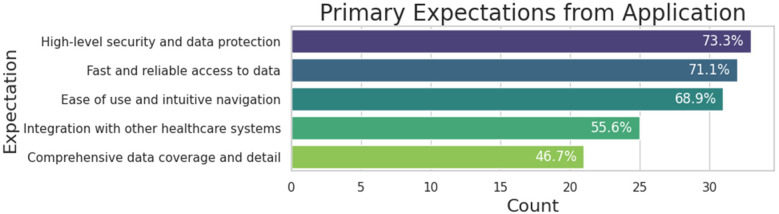
Participants’ expectations from the mobile application to manage their health.

Regarding data security, when asked “*How important is the security of medical data to you when using such applications?*”, 95% of participants stated that it is very to extremely important, while the remaining 5% considered it moderately important. This high concern for security is understandable given the sensitive nature of healthcare data. Figure 9 further illustrates participants' expectations for the mobile application, emphasizing the desire for a fast, reliable, and user-friendly interface that doesn't require significant effort to navigate.

### 4.2 User engagement metrics

**Figure 10:**
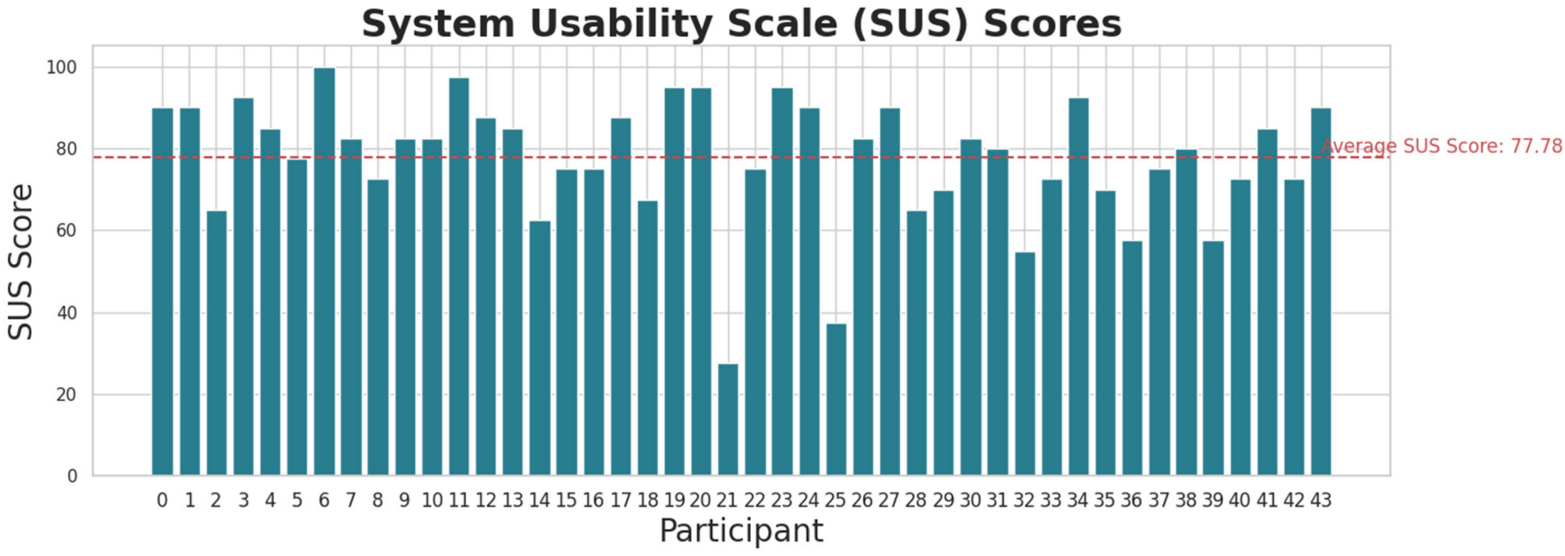
Participants’ score of the system usability questionnaire for our mobile application.

The usability assessment of our system yielded promising results, with the mobile application receiving an average score of 77.78 out of 100 in the System Usability Scale (SUS) questionnaire as illustrated in Figure 10. This score indicates that users generally found the application to be highly usable, though there remains potential for further enhancement. It is worth noting that participant 21 encountered technical difficulties with the patient summary translation feature, which may have contributed to their lower score and slightly impacted the overall average.

In addition to the SUS evaluation, we employed the Net Promoter Score (NPS) as a metric to gauge user satisfaction and likelihood of recommendation. Our application achieved an NPS of 40.91 from the −100 to 100 scale, which is a commendable result. This score suggests that a substantial portion of our participants would be willing to recommend the application to others, with promoters outnumbering detractors. While this score places our application in the “good” category, it also highlights the opportunity for further improvements to elevate the user experience to an “excellent” level.

For our last questionnaire in this section, the UEQ is structured to measure two main aspects of user experience:

1. Pragmatic Qualities: The first four questions focus on efficiency, ease of use, and other practical aspects of the application.
2. Hedonic Qualities: The subsequent four questions evaluate user satisfaction and the more emotional or pleasurable aspects of the user experience.

**Figure 11:**
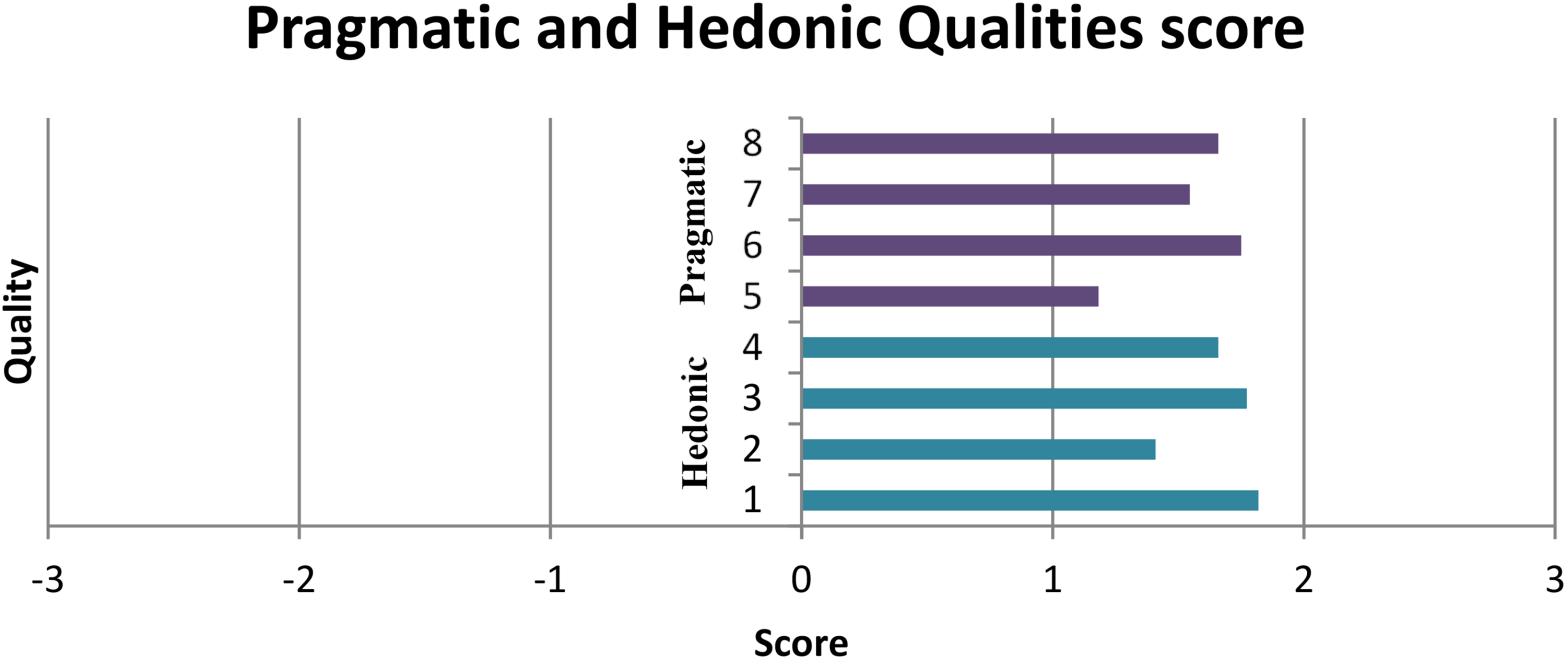
User experience qualities score. Values between −0.8 and 0.8 represent a neural evaluation of the corresponding scale, values greater than 0.8 represent a positive evaluation and values <−0.8 represent a negative evaluation. The range of the scales is between −3 (horribly bad) and +3 (extremely good). But in real applications in general only values in a restricted range will be observed. It is due to the calculation of means over a range of different people with different opinions and answer tendencies extremely unlikely to observe values above +2 or below −2.

Figure 11 presents a comprehensive view of the scores for all items, and the results are notably positive. With an overall score of 1.60 (calculated as the average of all values shown in Figure 11), the application is categorized as “excellent” in terms of user experience. This high score indicates that users found the application both practically efficient and enjoyable to use. To further validate these results, the questionnaire provides consistency estimates with the pragmatic values score to be 0.91 indicating an extremely important level of confidence in the results related to the application's practical aspects. The hedonic Qualities score of 0.72, while it is lower than the pragmatic score, still it is considered high. This suggests a satisfactory level of agreement among users regarding the application's satisfaction and pleasure-related aspects, albeit with slightly more variability in responses compared to the pragmatic qualities. These results collectively give an incredibly positive picture of the application's user experience. The high scores across both pragmatic and hedonic qualities, coupled with strong consistency estimates, suggest that the application successfully meets user needs in terms of functionality and enjoyability.

### 4.3 Performance metrics

The performance metrics of our application reveal promising results across various key indicators, though it is important to note that these were obtained in a testing environment with limited resources, as the OpenNCP server was not yet in live production. The translation of patient summaries, a critical feature of the system, demonstrated a mean response time of 17,511 milliseconds (approximately 17 s). While this duration may seem lengthy, it is crucial to consider the context of our testing environment. The response time is expected to improve significantly once the system is deployed in a live production environment with full resources allocated.

In terms of Smart Health Link (SHL) generation, the system exhibited exceptional speed, with a mean response time of just 253 milliseconds. This near-instantaneous generation of SHLs facilitates smooth and rapid sharing of health information, a crucial aspect for both patients and healthcare providers.

The reliability of the translation feature is evidenced by the high success rate, with 44 successful translations out of 45 attempts. Only one translation failed, indicating a 97.8% success rate, which is commendable for a complex task involving medical terminology and diverse language pairs, especially in a testing environment. All 45 attempts to generate SHLs were successful, demonstrating the robustness of this feature. This 100% success rate in SHL generation underscores the application's dependability in creating secure, shareable health information links. Regarding the validation of translated patient summaries against the CDA, the results were also positive. Out of 45 translated summaries, all passed the validation process, though 5 summaries triggered minor warnings due to OpenNCP version issues. Overall, these performance metrics indicate a promising system with strong potential, particularly in its core functionalities of translation and SHL generation.

### 4.4 User feedback

**Figure 12:**
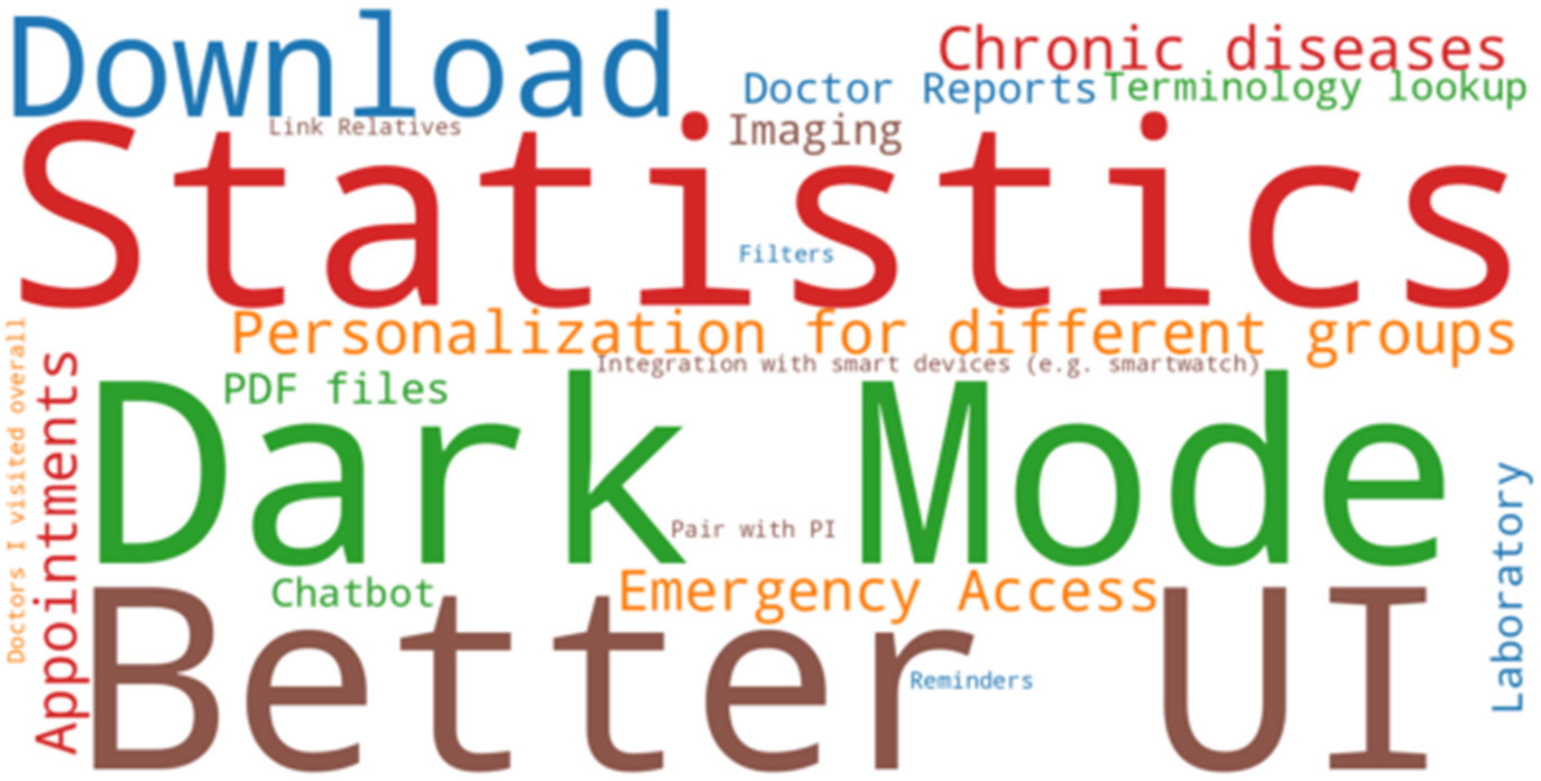
Word cloud of suggested features. Bigger font size means that more participants suggested that feature.

In the last two questions, we aimed to gather additional feedback from participants without restricting them to predefined options. Specifically, we asked what features they would like to see and invited any comments they had about the application. Figure 12 highlights the most requested features, with larger font sizes indicating higher demand. The top three features were Dark Mode, an Improved UI, and Statistics.

Currently, our application is designed with a Light Theme palette, but it appears that many participants prefer the option to switch to Dark Mode, likely for aesthetic reasons or improved eye comfort. Additionally, users suggested modernizing the user interface, as they found the current design somewhat outdated. The request for “Statistics” refers to a feature where the application could analyze a patient's summary and generate reports on their overall health. This would help condense large amounts of patient data into key insights and provide a more accessible overview of important information.

Participants also suggested the ability to download patient summaries, which could be useful for printing or sharing. Another recommendation was to personalize the app for different user groups, such as those with chronic diseases or older adults, as these groups have specific needs. For instance, we could simplify navigation for elderly users by reducing the number of clicks required to access resources. For chronic disease patients, we could create a dedicated section that focuses on medical data relevant to their condition. Finally, two additional notable features that were mentioned, which are already in our future development plans, include electronic appointment scheduling to improve communication between healthcare providers and patients, and a chatbot assistant to help users find resources more easily.

Although the last question was optional, we were pleased to see that nearly all participants provided feedback. Overall, many users expressed satisfaction with the application, leaving comments such as “Spectacular!!”, “Excellent”, “Very good job”, and “Amazing work so far”. Additionally, participants 40 and 43 highlighted the app's usefulness in facilitating communication between patients and doctors, making it easier to share medical data, prepare for visits, and ultimately save time by focusing on care rather than searching for patient records.

Participant 40 (Doctor): A very very important application and I am sure it is going to be super useful for people and doctors! Excellent job! Can't wait to use it and see it be used!

Participant 43 (Citizen): It is an extremely useful app that may solve a lot of problems and save a lot of time for both patients and doctors.

Regarding areas for improvement, we have selected three user comments that provide valuable insights. Participant 5 pointed out an oversight on our part: when translating the patient summary, all labels within the app are also translated. This can make it difficult for users to navigate if they are unfamiliar with the new language. We agree that the app's interface labels should remain in the user's native language, while only the medical information should be translated. Another important suggestion came from a paramedic, who expressed a need for access to the app when a user is unconscious in order to make informed decisions about their care. While privacy concerns prevent us from granting full access to a user's phone, smartphones do offer emergency features that allow limited access to specific parts of apps that users have pre-approved (55, 56). Implementing such a feature could address this concern.

Participant 5 (Paramedic): If I need to change the language of the application, when I change country, I would like to see at least the menu in my native language as well.

Participant 7 (Paramedic): I need the app when he is unconscious, when in critical situation you act fast and if anaphylaxis you fight it, also we need languages besides European countries, like Arabic, Japanese, South African etc.

Finally, Participant 27 provided constructive criticism regarding the UI. They suggested new design ideas and emphasized the importance of handling missing data and avoiding overlapping labels more carefully. Additionally, they noted that expanding language support beyond European languages is crucial for achieving a truly global reach.

Participant 27 (Citizen): The app needs a lot of work. The translation to Greek is not adaptive and the words don't even fit in some places and change line mid-word. The whole thing could be much professional. It looks like the frontend was done in 1 h. Even the tabs in the allergies are touching/overlapping each other. And most of the summary tabs were empty and I couldn't see examples. Also, the pictures in the Home bar are not HQ and the blurriness is visible. Nice concept, but the visuals of the app destroy the experience. Needs to be more user friendly (some old people will find it difficult to navigate).

## 5 Discussion

The results of our study provide valuable insights into the strengths and areas for improvement of our mobile application designed to facilitate multilingual access and sharing of health records. Overall, the feedback from participants was overwhelmingly positive, with many praising the app's user-friendliness and its potential to enhance communication between patients and healthcare providers. However, several constructive suggestions were made that highlight opportunities for further refinement. One of the most notable findings is the strong demand for a Dark Mode feature, which suggests that users are increasingly conscious of eye comfort and personalization options in mobile applications. This feature, along with a modernized user interface, was among the top requests from participants. The desire for a more contemporary design indicates that while the app's functionality is appreciated, aesthetics and user experience remain critical to its adoption.

Our study's findings agree with existing literature, highlighting the potential of mHealth apps. Participants in our pilot study frequently noted that such an application could greatly improve their health management and facilitate more effective communication with healthcare professionals, aligning with previous research that underscores the benefits of mobile health technologies for patient engagement and communication (17–20, 30, 31). However, participants also raised concerns about data security (21–23). Ensuring data security is universally recognized as a top priority for these applications, reflecting a common theme across literature that emphasizes the need for stringent privacy and security protocols in digital health solutions.

There is currently no universally accepted solution for supporting multilingual translations of medical records. Studies have shown that general machine translation tools are inadequate for medical records due to the complexity of medical vocabulary and syntax (57–59). Our system architecture addresses this issue by ensuring accurate translations of medical documents. This is achieved using OpenNCP, which effectively manages translation mappings, and all translations are rigorously reviewed by medical experts from each country. Our solution offers greater reliability and earns users' trust because the entire translation process is carefully supervised by medical experts. Unlike machine translation, which simply generates results based on algorithmic outputs, our approach ensures accuracy and quality through expert oversight.

Our solution serves as a foundational reference for developing multilingual functionalities in national health applications that manage EHRs. While countries like France (60), Germany (61), Portugal (62), Spain (63), Sweden (64), and the United Kingdom (65) have already a national health application, current literature indicates that these platforms lack features for multilingual patient summaries and medical record sharing. By using our system architecture and specifications as a guide, these applications can incorporate such features to significantly enhance the user experience and improve the management of citizens' health.

The integration of the SHL protocol enables precise, time-limited sharing of health data, offering a significant improvement over traditional EHR access models. Unlike conventional systems that often require sharing entire records, SHL allows patients to selectively share specific health resources, ensuring greater privacy and control. This approach fully complies with EHDS regulations by promoting interoperability and patient empowerment. Additionally, SHL eliminates reliance on specific accounts or platforms, as it operates through a secure passcode known only to the patient and their chosen recipients. This flexibility ensures that medical information is not confined to data silos or proprietary systems, facilitating seamless sharing across diverse healthcare environments while maintaining robust security and user autonomy.

In terms of usability, participants were generally satisfied with the app's performance, but some highlighted specific issues that need addressing. For instance, one participant pointed out that when translating patient summaries, all labels within the app are also translated, which can make navigation difficult if users are unfamiliar with the new language. This feedback suggests that while translation is a valuable feature, it should be applied selectively to avoid confusion, keeping interface labels in the user's native language while translating only medical information.

Additionally, a paramedic raised an important concern regarding access to patient data during emergencies when a user is unconscious. While privacy concerns prevent unrestricted access to a user's phone, this feedback underscores the need for emergency access features that allow healthcare professionals to retrieve critical information with user pre-authorization (55, 56). Such functionality could be vital in life-threatening situations where timely access to medical data is crucial.

A key aspect of these services is the availability of accurate and up-to-date patient data. This creates an added responsibility for healthcare professionals and systems, as they must ensure the synchronization of patient information from every doctor or provider involved in their care. Without updated data, there is a significant risk that healthcare decisions could be based on outdated or incomplete information, potentially overlooking new conditions or changes in the patient's health (66). This challenge is further amplified in interoperable systems, which require seamless communication across various platforms. In cases of conflicting information, there must be robust mechanisms in place to identify and resolve discrepancies, ensuring that the most accurate and current data is maintained.

Finally, one of the key takeaways from the pilot studies was the excitement and anticipation expressed by participants for the mobile application. Most agreed that it would be highly useful in their daily lives and were surprised that such simple, yet impactful features of translations and easy sharing of medical records are not already widely available. This feedback underscores the potential of our app to fill a significant gap in healthcare communication, particularly in cross-border scenarios where language barriers often hinder effective care.

For future work, we plan to upgrade the SHL to a new standard called Verifiable Health Links (67). This enhanced version will follow all the existing SHL protocol specifications but will add an extra layer of security and authenticity by incorporating a digital certificate to sign the links. Additionally, to create a more comprehensive mobile companion, we aim to implement several new features in our application. These include electronic appointment scheduling, biometric data collection through smart devices, an emergency button for quickly notifying emergency contacts, and the development of an online coach that provides patients with personalized smart interventions. All these are emerging eHealth features that have been promoted over the last few years (9).

## 6 Conclusions

Our study demonstrates that there is significant potential for mobile applications like ours to improve healthcare accessibility and communication across borders by enabling patients to control and share their medical data in multiple languages. The positive feedback from participants highlights that our solution addresses key issues in healthcare information management, particularly in overcoming language barriers and facilitating seamless sharing of health records.

However, there are areas where further development is needed. Enhancing personalization options such as Dark Mode and modernizing the user interface will improve user experience. Additionally, implementing advanced features like health statistics reporting and emergency access mechanisms will make the app more versatile and useful in critical situations. Expanding language support beyond Europe will also be essential for achieving global reach and ensuring that patients worldwide can benefit from this technology. By addressing these areas for improvement, we can continue to refine our solution and contribute meaningfully to the ongoing development of patient-centric healthcare systems.

In conclusion, our mobile application has shown great promise in bridging gaps in healthcare communication and accessibility. With continued enhancements based on user feedback, it has the potential to become an indispensable tool for both patients and healthcare providers globally.

## Data Availability

The datasets presented in this article are not readily available because data collected are only to generate statistics and find trends. Requests to access the datasets should be directed to Theodoros Solomou, tsolom01@ucy.ac.cy.
