# Supplementary material for: Bridging language barriers in healthcare: a patient-centric mobile app for multilingual health record access and sharing
Source: Front Digit Health. 2025 Feb 18;7:1542485. doi: 10.3389/fdgth.2025.1542485 (PMC11876183; doi:10.3389/fdgth.2025.1542485)
Supplement: Supplementary file 1 [file Datasheet1.pdf]

Supplementary Material

System Usability Questionnaire

1. I think that I would like to use this system frequently

Strongly Disagree 1 2 3 4 5 Strongly Agree

2. I found the system unnecessarily complex

Strongly Disagree 1 2 3 4 5 Strongly Agree

3. I thought the system was easy to use

Strongly Disagree 1 2 3 4 5 Strongly Agree

4. I think that I would need the support of a technical person to be able to use this system

Strongly Disagree 1 2 3 4 5 Strongly Agree

5. I found the various functions in this system were well integrated

Strongly Disagree 1 2 3 4 5 Strongly Agree

6. I thought there was too much inconsistency in this system

Strongly Disagree 1 2 3 4 5 Strongly Agree

7. I would imagine that most people would learn to use this system very quickly

Strongly Disagree 1 2 3 4 5 Strongly Agree

8. I found the system very cumbersome to use

Strongly Disagree 1 2 3 4 5 Strongly Agree

9. I felt very confident using the system

Strongly Disagree 1 2 3 4 5 Strongly Agree

10. I needed to learn a lot of things before I could get going with this system

Strongly Disagree 1 2 3 4 5 Strongly Agree

Ease of Use and Usefulness Questionnaire

I can understand the terms and icons used by this module

Strongly Disagree 1 2 3 4 5 6 7 Strongly Agree

The module allows me to learn how to use it without a great deal of thought

Strongly Disagree	1	2	3	4	5	6	7	Strongly Agree

When I use this module, it always does what I want it to do

Strongly Disagree	1	2	3	4	5	6	7	Strongly Agree

Features and information are always where I expect them to be

Strongly Disagree	1	2	3	4	5	6	7	Strongly Agree

The module is right for me

Strongly Disagree	1	2	3	4	5	6	7	Strongly Agree

I would like to be a regular user of this module

Strongly Disagree	1	2	3	4	5	6	7	Strongly Agree

The module has exactly the features I need

Strongly Disagree	1	2	3	4	5	6	7	Strongly Agree

Trying out the module was exciting

Strongly Disagree	1	2	3	4	5	6	7	Strongly Agree

The module has interesting new features

Strongly Disagree	1	2	3	4	5	6	7	Strongly Agree

I like the look of the module

Strongly Disagree	1	2	3	4	5	6	7	Strongly Agree

The module looks like it is part of a high-quality mobile application

Strongly Disagree	1	2	3	4	5	6	7	Strongly Agree

User experience

Decide as spontaneously as possible which of the following opposing terms better describes the application. There is no "right" or "wrong" answer. Only your personal opinion counts!

Obstructive	○	○	○	○	○	○	○	Supportive
Complicated	○	○	○	○	○	○	○	Easy
Inefficient	○	○	○	○	○	○	○	Efficient
Confusing	○	○	○	○	○	○	○	Clear
Boring	○	○	○	○	○	○	○	Exciting
Not interesting	○	○	○	○	○	○	○	Interesting
Conventional	○	○	○	○	○	○	○	Inventive
Usual	○	○	○	○	○	○	○	Leading edge
